# Two Decades of Disease Evolution and Biomarker-Guided Clinical Decision Making in Metastatic Prostate Cancer

**DOI:** 10.3390/ijms26157593

**Published:** 2025-08-06

**Authors:** Tatiana Erazo, Enrico Moiso, Omer Aras, Howard I. Scher

**Affiliations:** 1 Translational Partnership Program, Memorial Sloan Kettering Cancer Center (MSK), New York, NY 10065, USA; erazoani@mskcc.org (T.E.); moisoe@mskcc.org (E.M.); 2 Department of Radiology, Memorial Sloan Kettering Cancer Center, New York, NY 10065, USA; araso@mskcc.org

**Keywords:** metastatic castration-resistant prostate cancer (mCRPC), longitudinal genomic profiling, liquid biopsy, AR amplification, bipolar androgen therapy (BAT), tumor evolution

## Abstract

Despite significant advances in prostate cancer treatment over the past two decades, metastatic castration-resistant prostate cancer (mCRPC) remains incurable. We present the case of a patient with aggressive prostate cancer diagnosed 20 years ago, underscoring the value of longitudinal genomic profiling and advanced imaging to guide clinical decisions. After multiple treatment failures, genomic analyses of tissue and liquid biopsies revealed dynamic changes in tumor biology and the emergence of resistance mechanisms, particularly AR amplification, identified with a liquid biopsy test and validated by [^18^F]-FDHT PET scan. This finding guided treatment with bipolar androgen therapy (BAT), which achieved a dramatic clinical response, reduced AR expression, improved symptoms, and restored sensitivity to enzalutamide. This case exemplifies the utility of serial liquid biopsies in uncovering mechanisms of tumor evolution and resistance, and the crucial role of cutting-edge diagnostics in personalized cancer treatment.

## 1. Introduction

In the past two decades, the treatment landscape for prostate cancer has undergone a marked transformation that has significantly improved patient outcomes. This includes the regulatory approval of 16 new life-prolonging treatments, such as next-generation androgen receptor (AR) signaling inhibitors, PARP inhibitors, radioligand therapies, and other targeted approaches [1,2], which together have increased the 5-year relative survival rate from 68% in 1975 to 97% today for localized disease [3]. However, despite these advances, metastatic castration-resistant prostate cancer (mCRPC) is rarely curable, with a median survival of less than three years [4].

Overall AR signaling inhibitors (ARSis) remain the mainstay of treatment of mCRPC, but more recently their use has been expanded to earlier stages of the disease [5,6], a transition that has led to longer exposure of more potent inhibitors, resulting in the emergence of new resistance mechanisms and more complex tumor evolutions that include lineage plasticity [7,8,9].

Fortunately, in parallel, significant advances in biomarker-focused strategies have deepened our understanding of the disease, leveraging insights from genomics, clonal evolution, and the tumor microenvironment. However, given the predominance of osseus spread in the mCRPC state [10], tissue biopsies, still considered to be the gold standard to molecularly profile a patient’s disease, often do not provide sufficient material for comprehensive analysis that fully captures the complex intra-and interlesional biologic heterogeneity [11,12].

To address this, liquid biopsies have emerged as an alternative for real-time disease monitoring through the analysis of circulating tumor DNA (ctDNA), which has demonstrated the presence of multiple subclones with distinct genomic features [13]. Unlike single-site metastatic biopsies, which contribute only a small fraction of the total ctDNA, serial blood sampling with ctDNA provides higher resolution of treatment-associated dynamics [14] to assess treatment efficacy, disease progression, and changes in tumor biology [15,16,17].

Noninvasive imaging has also evolved significantly during this time, further revolutionizing cancer diagnosis and treatment. Traditional modalities such as CT and bone scans are now complemented by multiparametric whole-body magnetic resonance imaging (WB-MRI), which better identifies areas of tumor spread; FDG PET-CT, available since the 1990s, which identifies active and non-active areas of disease spread [18]. Later, PSMA PET-targeted imaging significantly improved the detection of disease, enabling the development of radioligand therapies such as 177Lu-PSMA-617 [2,19,20] and [^18^F]-FDHT PET to quantify AR expression levels in individual lesions [21,22]. The combined result is a marked improvement in determining the disease presence and extent, therapeutic efficacy, and patient selection for targeted therapies [23]. In prostate cancer specifically, these advanced imaging modalities have improved decision-making accuracy by providing deeper insights into the complex heterogeneity that characterizes individual tumors as they evolve through treatment response and disease progression.

In this report, we describe an ongoing 20-plus-year case of a prostate cancer patient who presented with aggressive disease, highlighting our evolving ability to understand and manage his disease as improved imaging modalities and molecular profiling of tissue and blood became available. Specifically, a liquid biopsy analysis identified AR amplification as the underlying resistance mechanism, which was subsequently validated by [^18^F}-FHDT PET imaging. This led to bipolar androgen therapy (BAT), which is a high-testosterone therapeutic approach that successfully reduced AR expression, disease burden, improved anemia and symptoms, and restored sensitivity to the AR antagonist enzalutamide. This case illustrates both the utility of longitudinal genomic profiling in guiding clinical decisions and the efficacy of BAT to overcome AR amplification-driven resistance.

## 2. Results

### 2.1. Clinical Case

The patient is currently a 72-year-old man, initially diagnosed with prostate adenocarcinoma in 2004, at the age of 52, following a screening test that showed a prostate-specific antigen (PSA) level of 27.06 ng/mL. No metastatic disease was detected in the preoperative staging evaluation, confirmed at radical retropubic prostatectomy (RP), which showed stage IV disease (pT4N0M1b) with a Gleason score of 9 (4 + 5), with right extracapsular extension and invasion of the right seminal vesicle and bladder neck, indicating a high risk of local and systemic recurrence. He subsequently received postoperative hormone therapy for one month, which resulted in an undetectable PSA two months later. After a slight PSA rise in 2005, and his positive bladder neck margins, radiotherapy was administered at 7200 cGy in 40 fractions.

The patient remained asymptomatic until 2008, when he experienced a biochemical recurrence; at a time when next-generation imaging was not standard practice, and bone scans, despite their limited sensitivity, were the primary tool for staging and monitoring biochemical recurrence. At this point, to better assess and inform management, FDG PET-CT and WB-MRI were performed, which showed a single FDG-avid osseous lesion and an avid inguinal lymph node, while concurrent bone scans failed to detect any osseous spread. As a result, he was treated with a single fraction of image-guided radiation therapy (IGRT) at a dose of 24 Gy and three months of androgen deprivation therapy (ADT), after which his PSA remained undetectable until 2014.

Again in 2014, after another biochemical recurrence, a radionuclide bone scan showed no evidence of disease, while FDG PET-CT and WB-MRI identified multiple new FDG-avid bone metastases and an avid inguinal lymph node. The patient was treated with ADT for 3 months, resulting in a PSA nadir of <0.1 ng/mL, which remained stable for over 12 months. Together, the detection capabilities of FDG PET-CT and WB-MRI, compared with traditional bone scan imaging, have improved the identification of bone and lymph node metastases in prostate cancer.

**Figure 1:**
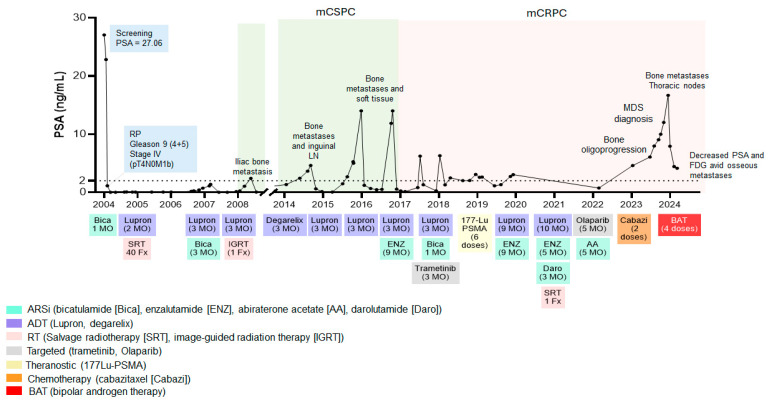
PSA kinetics throughout the patient’s clinical course and therapeutic interventions. PSA levels over time in relation to disease progression and sequential treatment regimens: ARSi (androgen receptor signaling inhibitor, turquoise), ADT (androgen deprivation therapy, purple), RT (radiotherapy, pink), targeted therapy (gray), theranostics (yellow), chemotherapy (orange), and BAT (red).

In 2016, another biochemical recurrence occurred and series of FDG PET-CT and WB-MRI imaging again showed further osseous spread and new soft tissue disease between the bladder wall and right pubic bone, then controlled for a period of 9 months with ADT, following which, in 2017 and after multiple PSA fluctuations, the disease became castration-resistant, so he was started on enzalutamide as first-line treatment for 6 months and Lupron for 3 months in 2017 and 2018 (Figure 1).

**Figure 2:**
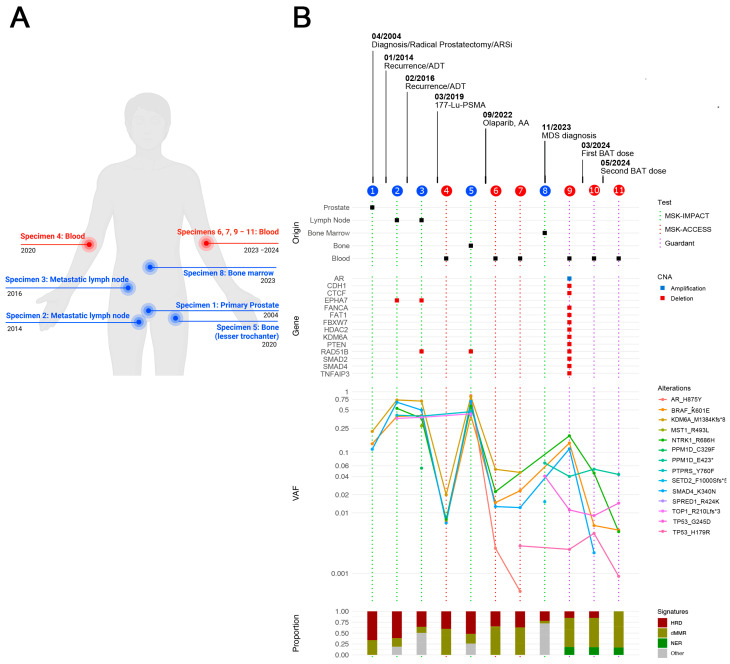
Longitudinal molecular profiling and mutation analysis over two decades of disease course using the MSK-IMPACT, MSK-ACCESS, and Guardant360 platforms. (**A**) Schematic illustrating the origin of samples collected at multiple time points throughout the course of the disease. Created in BioRender (https://app.biorender.com/, accessed on 3 August 2025). (**B**) Comprehensive genomic profiling. The upper panel shows the origin, year of collection, and clinical event associated with each biopsy. Tissue biopsies were analyzed using MSK-IMPACT (green dashes) and cfDNA was assessed via MSK-ACCESS (red dashes) or the Guardant360 assay (purple dashes). Second panel: copy number alterations (CNAs) are depicted with gene amplifications in blue and deletions in red. Third panel: variant allele frequencies (VAFs) of 14 unique mutations identified at different sampling sites are also shown. The bottom panel presents a mutational signature analysis demonstrating enrichment of pathways, including homologous recombination deficiency (HRD), DNA mismatch repair deficiency (dMMR), and nucleotide excision repair (NER), that correlate with changes in cancer biology.

Around this time, comprehensive genomic profiling was becoming an essential tool to personalize cancer management. Unfortunately, this approach was not available during the early stages of his disease and only became accessible at our institution in 2015. Given the continued disease progression after multiple lines of therapy, next-generation sequencing (NGS) was performed using biopsies collected at various time points throughout the course of the disease (Figure 2A).

The initial molecular analysis was performed on his primary prostate tumor, collected prior to the initiation of systemic treatment and at the time of radical prostatectomy in 2004, using the first generation of the MSK-Integrated Mutation Profiling of Actionable Cancer Targets (MSK-IMPACT) panel developed at Memorial Sloan Kettering Cancer Center, which subsequently received FDA clearance in 2017 [24].

Additional tumor samples from metastatic lymph nodes collected in 2015 and 2016 were also analyzed to identify actionable genomic alterations. In 2018, since *BRAF^K601E^* was the only clinically actionable mutation identified across all samples, the patient received trametinib, an FDA-approved MEK inhibitor, that has shown clinical benefit in advanced solid tumors with *BRAF^K601E^* mutation [25]. Treatment was discontinued after three months due to lack of response.

Although 177Lutetium PSMA received FDA approval in 2022 [26], several studies conducted between 2015 and 2018 demonstrated its potential as a treatment for patients with mCRPC, particularly those who had exhausted conventional therapies, showing encouraging biochemical and radiographic response rates, as well as good tolerability [27,28,29]. Given this growing evidence and the limited treatment options at the time, the patient received six cycles of 177Lu-PSMA therapy in 2019. He reached a PSA nadir < 1 ng/mL one month after 177Lu-PSMA treatment, but levels began rising after 6 months.

Next, the patient received a third-line treatment with Olaparib, abiraterone acetate, and prednisone. Despite initial biochemical response, treatment was discontinued after 5 months due to bone oligoprogression.

In November 2023, a bone marrow aspiration biopsy revealed myelodysplastic syndrome (MDS) and clonal cytopenia, which are known adverse effects of theranostic therapy [30,31]. Due to the indolent nature of MDS, active surveillance was chosen, prioritizing treatment of the progressive prostate cancer (Figure 1). At this point, chemotherapy became necessary; however, docetaxel was contraindicated due to bone marrow involvement, prompting treatment with cabazitaxel. Nevertheless, cabazitaxel was discontinued after only two cycles due to neutropenia and increasingly fragile clinical condition.

In parallel, circulating cell-free DNA (cfDNA) analyses from peripheral blood samples collected in 2020 and 2023 were performed using Circulating cfDNA to Examine Somatic Status (MSK-ACCESS), which is a 129 key cancer-associated gene panel also developed at our institution and approved for clinical use in 2019 [32]. Genomic analysis identified 14 unique mutations across all specimens. Among these, three somatic mutations (*BRAF^K601E^*, *KDM6A^M1384fs^*, and *SMAD4^K340N^*) were consistently detected in the primary tumor, metastatic lesions, and cfDNA samples collected throughout disease progression (Figure 2B).

In 2023, genomic analysis of a bone marrow biopsy was performed using the MSK-IMPACT Heme panel, along with cfDNA analysis using the Guardant360 assay, which revealed high AR amplification at 3.96 copies. Interestingly, concurrent MSK-IMPACT Heme testing did not detect AR amplification. This discrepancy likely reflects tumor heterogeneity in mCRPC. It is plausible that AR-amplified tumor clones were predominantly confined to bone and thoracic lymph node metastases, while remaining below the detection threshold in sampled bone marrow tissue.

**Figure 3:**
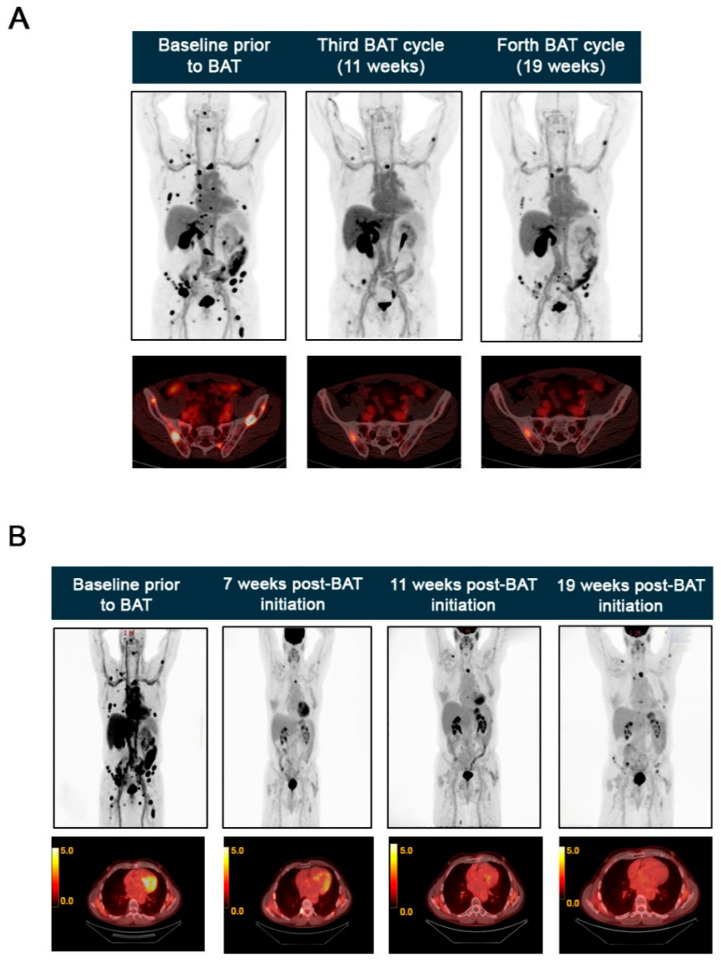
Multi-modal PET imaging demonstrates the therapeutic response to BAT. (**A**) Baseline [^18^F]-FDHT PET scan (left) demonstrates high AR expression in multiple metastatic lesions. Follow-up [^18^F]-FDHT PET scans at cycle 3 (11 weeks, center) and cycle 4 (19 weeks, right) of BAT treatment showing near complete resolution of previously identified metastatic lesions. (**B**) Serial FDG PET-CT scans showing response to BAT. Baseline pre-treatment FDG PET scan showing increased FDG uptake in multiple bones and thoracic nodes. Follow-up FDG PET-CT scans at the second cycle (7 weeks), third cycle (11 weeks), and fourth cycle (19 weeks) of BAT treatment, respectively, showing significantly lower FDG uptake compared to the pre-treatment scan, confirming continued treatment response.

*NTRK1^R686H^* and *BRAF^K601E^* were the top somatic mutations identified by the Guardant360 assay, with variant allele fractions (VAFs) of 18.8% and 14.2%, respectively. *TP53^G245D^* was the only alteration detected by both platforms (Figure 3A).

To identify the molecular processes driving the changes in somatic mutations throughout tumor evolution and treatment, we performed COSMIC somatic signature analysis, which assigns mutational signatures to each individual sample. Figure 2B shows significant changes in signature profiles over time, including decreased homologous recombination deficiency (HRD) signature activity. In contrast, increased DNA mismatch repair (dMMR) signature activity, which, while a rare phenotype in prostate cancer, can be acquired during disease progression and is often associated with a higher mutational burden, typically associated with advanced-stage disease [33]. The nucleotide excision repair (NER) signature, another canonical DNA damage response (DDR) pathway, was significantly enriched during oligoprogression. We also observed an increase in the APOBEC-induced signature, which, as previously described, can be intermittently turned on and off by unknown mechanisms [34].

#### BAT Eliminates AR Amplification Detected by cfDNA Analysis

In 2024, a restaging FDG PET-CT scan showed further osseous spread involving the skull base, spine, thorax, pelvis, and extremities and mildly avid thoracic nodes. Given the AR amplification detected by the Guardant360 test; an AR-targeted imaging scan [^18^F]-FDHT PET was performed as part of an IRB-approved institutional research protocol. Although [^18^F]-FDHT PET is currently primarily used in research settings rather than as a routine clinical imaging procedure, it has been extensively validated as a reproducible method for in vivo AR imaging [21,35,36]. In this case, the scan confirmed the distribution of metastatic lesions with high AR expression (Figure 3A).

BAT has emerged as a promising approach for mCRPC patients with high AR expression and resistance to low testosterone levels [35]. Based on these findings, the patient was started on BAT, consisting of four intramuscular injections of 400 mg testosterone cypionate.

He subsequently demonstrated clinical improvement in energy and a PSA decline from 16.67 to 4.14 ng/mL, within 7 weeks of starting BAT treatment. FDG PET-CT scans performed in May, June, and August 2024, corresponding to the second cycle (7 weeks), third cycle (11 weeks), and fourth cycle (19 weeks) of BAT treatment, confirmed a dramatic therapeutic response. Overall, the intensity of FDG-avid osseous metastases decreased markedly, with several lesions showing activity below background, and the thoracic nodes were resolved. Only a few lesions remained unchanged (Figure 3B).

Two Guardant360 tests performed at the follow-up time points described above showed eradication of the AR amplification. cfDNA analysis also showed that the VAF of *NTRK1^R686H^* and *BRAF^K601E^* decreased to 0.5% (Figure 2). Furthermore, follow-up [^18^F]-FDHT PET scans revealed a marked reduction in tracer uptake in the third and fourth BAT cycles, confirming a decrease in AR expression in metastatic lesions that previously had high expression (Figure 3A).

Given the dramatic response to the BAT, ADT therapy with degarelix was restarted one month after the last testosterone cypionate injection. After two months on ADT, the patient was started on enzalutamide, which continues as his current therapeutic regimen.

## 3. Discussion

The present case report underscores the challenges of managing mCRPC following the development of resistance to multiple lines of treatment, including hormonal (bicalutamide, enzalutamide, and darolutamide), ADTs (Lupron and degarelix), targeted therapies (Olaparib, trametinib, and 177Lu-PSMA), and taxane-based therapies (cabazitaxel). This case also highlights the importance and value of longitudinal genomic profiling to identify changes in tumor biology and treatment resistance mechanisms, such as AR amplification, which occurs in response to prolonged exposure to ADT and affects up to 50% of mCRPC patients [36,37].

BAT is a therapeutic strategy for patients with mCRPC and elevated AR expression that involves the administration of high-dose testosterone to achieve supraphysiological concentrations, which subsequently restore AR and testosterone levels to near-normal ranges [35,38]. The Phase II RESTORE study demonstrated that BAT induced a 70% PSA response rate, with a median response duration of about 6 months, in patients who received enzalutamide rechallenge after BAT treatment, demonstrating BAT’s potential to resensitize tumors to AR-targeted therapies [39]. Moreover, the Phase II TRANSFORMER trial demonstrated superior progression-free survival with BAT compared to enzalutamide in patients with full-length AR expression [40].

The efficacy of BAT is postulated to be due to the induction of DNA double-strand breaks and the suppression of DNA repair gene expression [41]. Notably, patients with mutations in homologous recombination repair (HRR) genes and *TP53* have shown durable responses to BAT [42,43,44]. A combinatorial trial of BAT with DNA-repair inhibitor Olaparib (NCT03516812) demonstrated enhanced clinical activity, suggesting that BAT may induce HRD and sensitize cancer cells to PARP inhibition [45].

In this case, liquid biopsy tests revealed *TP53* mutations (G245D and H179R) and multiple alterations in the HRR gene, *ATM,* including codons R300H, D841fs, and I2701L, which could contribute to the patient’s dramatic response to BAT. While we are currently evaluating the response to rechallenge with enzalutamide, the characteristically limited progression-free survival with BAT treatment (<6 months) [39] indicates that alternative therapeutic approaches will likely be required in the near future. Considering the patient’s *TP53* mutation status and enrichment of the deficient NER mutational signature (previously linked to sensitivity to platinum-based therapy) [46], the combination of BAT with carboplatin, currently under evaluation in a phase II clinical trial (NCT03522064) [44], could represent a potential therapeutic strategy. However, prospective clinical trials are needed to establish optimal treatment sequencing and identify synergistic combination approaches in the post-BAT setting.

The patient’s limited response to trametinib monotherapy is consistent with previous reports and highlights the need for more effective targeted strategies [47,48]. Looking ahead, novel BRAF-targeted agents, such as Plixorafenib (NCT05503797) [49], are poised to expand therapeutic options for patients with *BRAF^K601E^*, potentially improving clinical outcomes where current approaches have failed.

The *AR^H875Y^* mutation affects the AR ligand-binding domain (LBD) and is present in 10–25% of mCRPC cases [50], conferring resistance to antiandrogen therapies such as bicalutamide, abiraterone, enzalutamide, or apalutamide [51]. Notably, both tissue and liquid biopsy testing detected the emergence of this alteration in 2020 following prolonged exposure to antiandrogens, which could be an additional resistance mechanism acquired during the disease evolution. While this alteration currently lacks approved targeted therapies, several AR degraders are in clinical development and could offer future therapeutic opportunities.

In this case, disease progression was characterized by dynamic changes in mutational signatures, offering insights into tumor biology and therapeutic vulnerabilities. Notably, during a key progression event in 2022, persistent HRD signature enrichment, along with preliminary data from the LuPARP trial (NCT03874884), showing that Olaparib enhances the efficacy of 177Lu-PSMA by inhibiting DNA repair, supported its use following radioligand therapy [52,53]. Although the response was brief, this case illustrates the clinical utility of integrating genomic signatures in advanced prostate cancer.

The emergence of APOBEC-associated and DNA repair-related signatures (e.g., NER and dMMR) reflects increased tumor heterogeneity and suggests an adaptive response to therapeutic pressure during metastasis. Tumors with DNA repair defects often compensate by relying on alternative pathways to maintain genomic instability while avoiding lethal DNA damage [54,55]. These observations underscore how dynamic mutational processes can reflect both the consequences and potential drivers of resistance.

In conclusion, this case illustrates how advancements in biomarker research, including clinical-grade comprehensive genomic profiling tests and next-generation imaging modalities, have transformed cancer care and are critical for understanding disease biology and guiding clinical decisions. Longitudinal genomic analysis proves essential for understanding tumor evolution and identifying resistance mechanisms in heavily pretreated mCRPC. Finally, this case supports the efficacy of BAT in achieving biochemical and clinical response in a challenging patient population.

## 4. Materials and Methods

### 4.1. Tumor Genomic Profiling with MSK-IMPACT

Tumor DNA was extracted from formalin-fixed, paraffin-embedded (FFPE) biopsy samples, while matched normal DNA was isolated from peripheral blood mononuclear cells. DNA input was optimized at 50–250 ng per sample, depending on sample availability. MSK-IMPACT identifies somatic mutations, copy number alterations (CNAs), and structural variants (SV) in 505 cancer-related genes by broad hybrid capture next-generation sequencing [56]. Both tumor and normal captured libraries were sequenced on Illumina HiSeq 2500 or NovaSeq 6000 platforms.

### 4.2. ctDNA Profiling

Peripheral venous blood was collected in two 10 mL Cell-Free DNA BCT® tubes (Streck, La Vista, NE, USA) tubes and processed at MSKCC for MSK-ACCESS or shipped to Guardant Health (Redwood City, CA, USA) for Guardant360 testing at room temperature. Whole blood was centrifuged at 800× *g* for 10 min at room temperature, and plasma and buffy coat were separated and stored at −80 °C until DNA isolation. cfDNA was isolated using the QIAmp Circulating Nucleic Acid Kit (Qiagen; Germantown, MD, USA) according to the manufacturer’s protocol. Extracted gDNA was quantified using NanoDrop (Thermo Scientific; Waltham, MA, USA), and 10 to 30 ng of plasma cfDNA were used for NGS. Both MSK-ACCESS and Guardant360 panels utilize targeted hybrid capture technology with genomic libraries constructed from cfDNA. MSK-ACCESS detects all classes of somatic alterations in 129 cancer-related genes [32]. The Guardant360 cfDNA assay identifies single-nucleotide variants (SNVs) in 73 genes, insertions/deletions (indels) in 23 genes, CNAs in 18 genes, and gene fusions in 6 genes [57]. Samples were sequenced on Illumina HiSeq 2500 or NovaSeq 6000 instruments.

### 4.3. Mutational Signatures Analysis

Catalogue of Somatic Mutations in Cancer (COSMIC) signatures database version 2 (https://cancer.sanger.ac.uk/cosmic/signatures_v2, accessed on 29 May 2025) [58] and the MSK-TempoSig algorithm (https://github.com/mskcc/temposig, accessed on 29 May 2025) were used to infer mutational signatures on sequencing data from MSK-IMPACT, MSK-ACCESS and Guardant360 panels ran on tumor tissues and blood samples.

## Data Availability

The original contributions presented in this study are included in the article. Further inquiries can be directed to the corresponding author.

## Abbreviations

The following abbreviations are used in this manuscript:

mCRPC	Metastatic castration-resistant prostate cancer
AR	Androgen receptor
PSA	Prostate-specific antigen
BAT	Bipolar androgen therapy
ADT	Androgen deprivation therapy
ARSi	Androgen receptor signaling inhibitors
177Lu-PSMA	177Lutetium PSMA
SRT	Salvage radiotherapy
ctDNA	Circulating tumor DNA
cfDNA	Cell-free DNA
NGS	Next-generation sequencing
MSK-IMPACT	MSK-Integrated Mutation Profiling of Actionable Cancer Targets
MSK-ACCESS	Circulating cfDNA to Examine Somatic Status
VAF	Variant allele frequency
CNA	Copy number alteration
WB-MRI	Whole-body magnetic resonance imaging
IGRT	Image-guided radiation therapy
HRD	Homologous recombination deficiency
dMMR	DNA mismatch repair
NER	Nucleotide excision repair
